# Supplementary material for: Targeting codon 158 p53-mutant cancers via the induction of p53 acetylation
Source: Nat Commun. 2020 Apr 29;11:2086. doi: 10.1038/s41467-020-15608-y (PMC7190866; doi:10.1038/s41467-020-15608-y)
Supplement: Supplementary file 1 — Supplementary Information [file 41467_2020_15608_MOESM1_ESM.pdf]

Targeting codon 158 p53-mutant cancers via the induction of p53 acetylation

Kong *et. al.*

Supplementary Figure 1

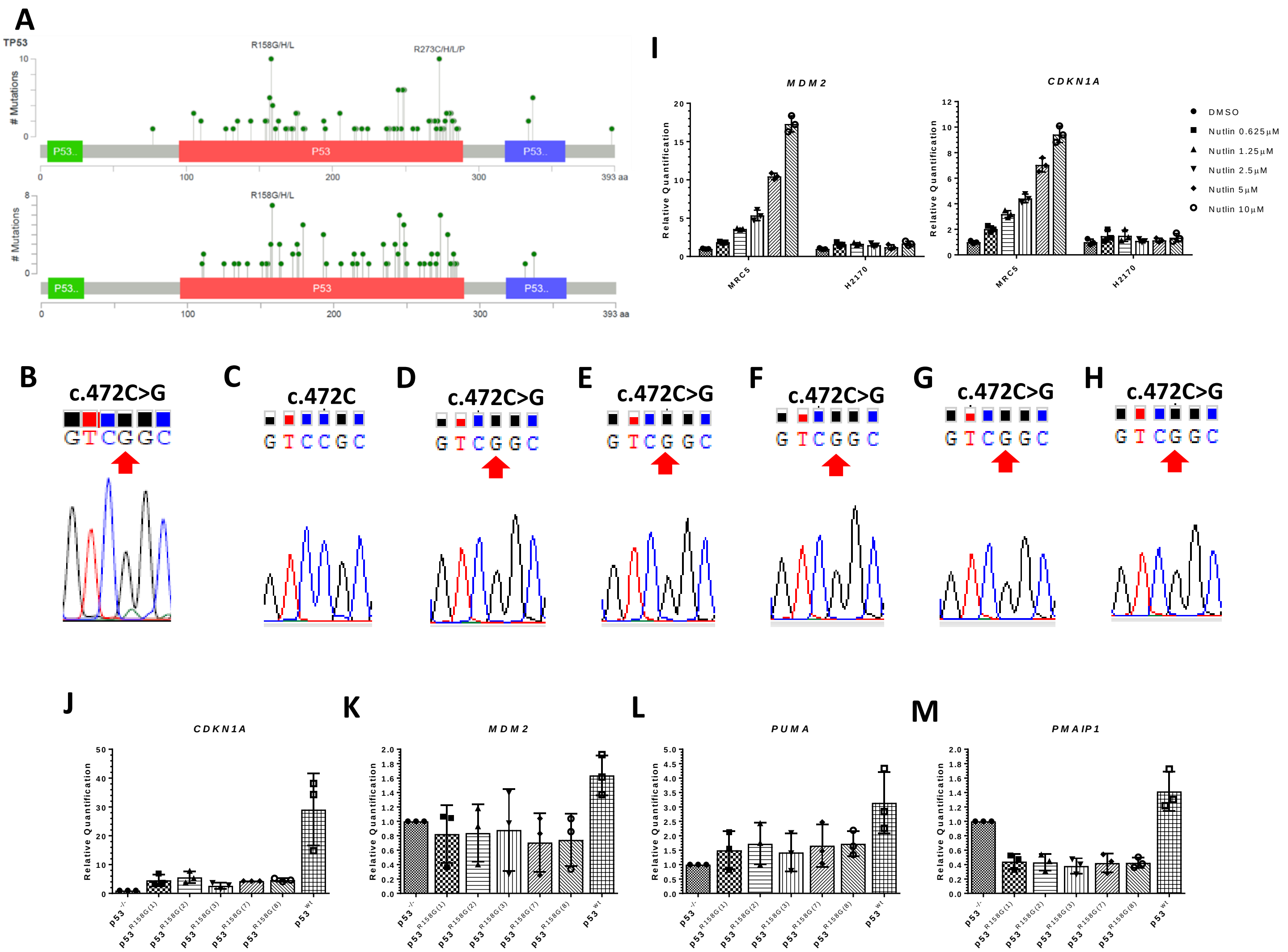

Supplementary Figure 1:
Mutation at codon 158 is a GOF p53 isoform (continue from Figure 1).
(A) Lolliplots showing TCGA mutational profile of *TP53* gene in lung squamous cell carcinoma (LUSC, top) and lung adenocarcinoma (LUAD, bottom).
(B-H) Sanger sequencing of *TP53* gene from cDNA of H2170 cells (B), Calu-1 wtp53 clone (C), and mutp53^{R158G} clone 1 (D), clone 2 (E), clone 3 (F), clone 7 (G) and clone 8 (H).
(I) RT-qPCR quantification of *MDM2* and *CDKN1A* mRNA expression in MRC5 and H2170 cell lines 48 hours after vehicle- or Nutlin-3a treatment.
(J-M) RT-qPCR analyses of the basal mRNA levels of *CDKN1A* (J), *MDM2* (K), *PUMA* (L) and *PMAIP1* (*Noxa*) (M) in various p53 clones (p53$^{-/-}$, p53^{wt}, p53^{R158G}). Data are expressed as average relative quantification ± SD (n = 3 independent experiments).

Supplementary Figure 2

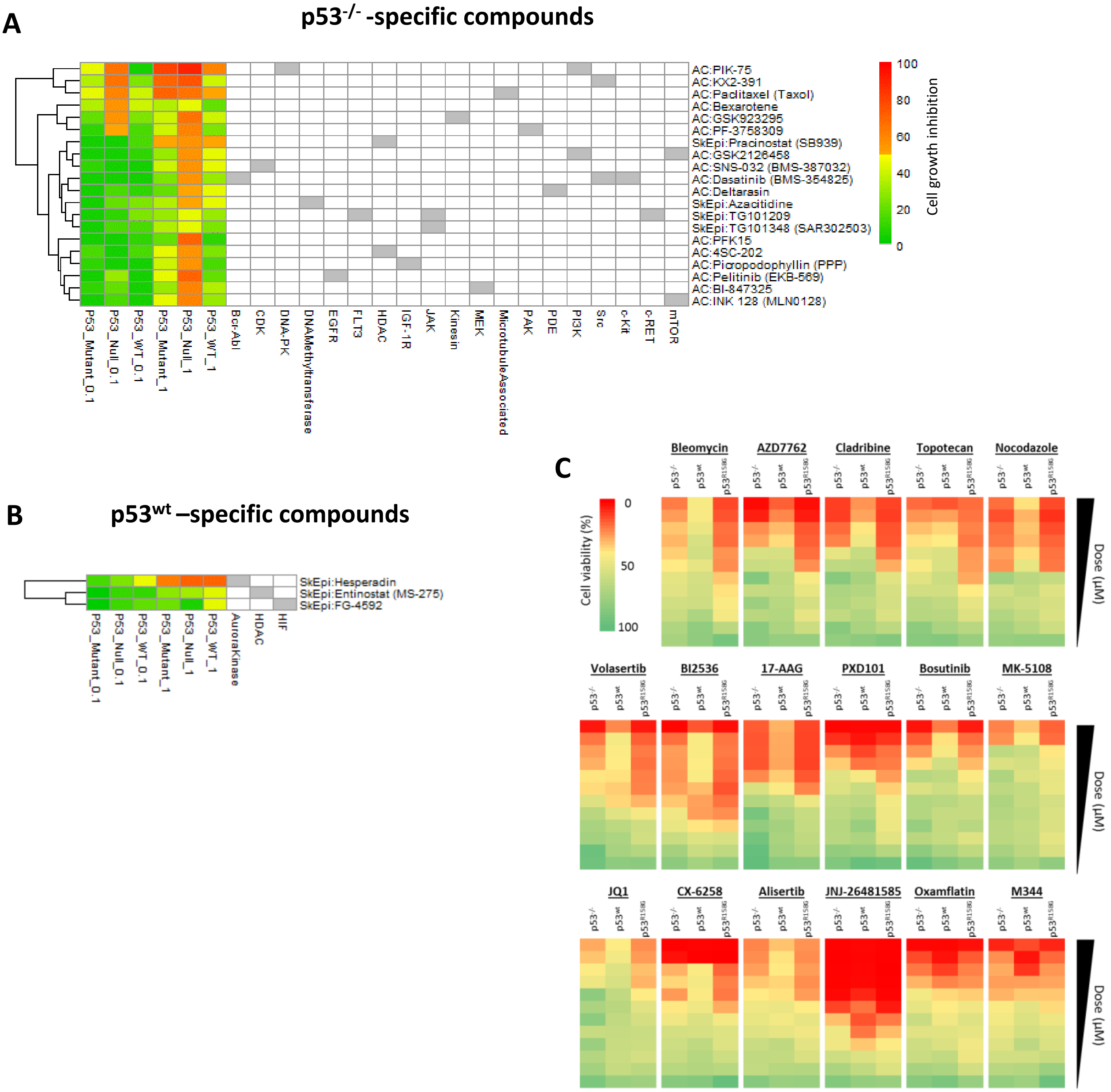

Supplementary Figure 2:
High-content screening of anti-cancer compounds and epigenetic modulators on p53 clones (continue from Figure 2).
(A-B) High-content screening of anti-cancer compounds and epigenetic modulators on isogenic p53 clones. The efficacy of the tested compounds was quantified relative to the mean viability of vehicle-treated cells (384-well format, in triplicates) (*n* = 1 independent experiment). Heatmap shows compounds filtered for specificity to p53$^{-/-}$ cells (A) or p53^{wt} cells (B) (growth inhibition > 50% in specific cell type).
(C) Validation of *in vitro* activity of selected compounds against p53 clones (p53$^{-/-}$, p53^{wt}, p53^{R158G}) in dose-response (384-well format, in triplicates). Data are presented as mean cell viability (*n* = 3 independent experiments).

Supplementary Figure 3

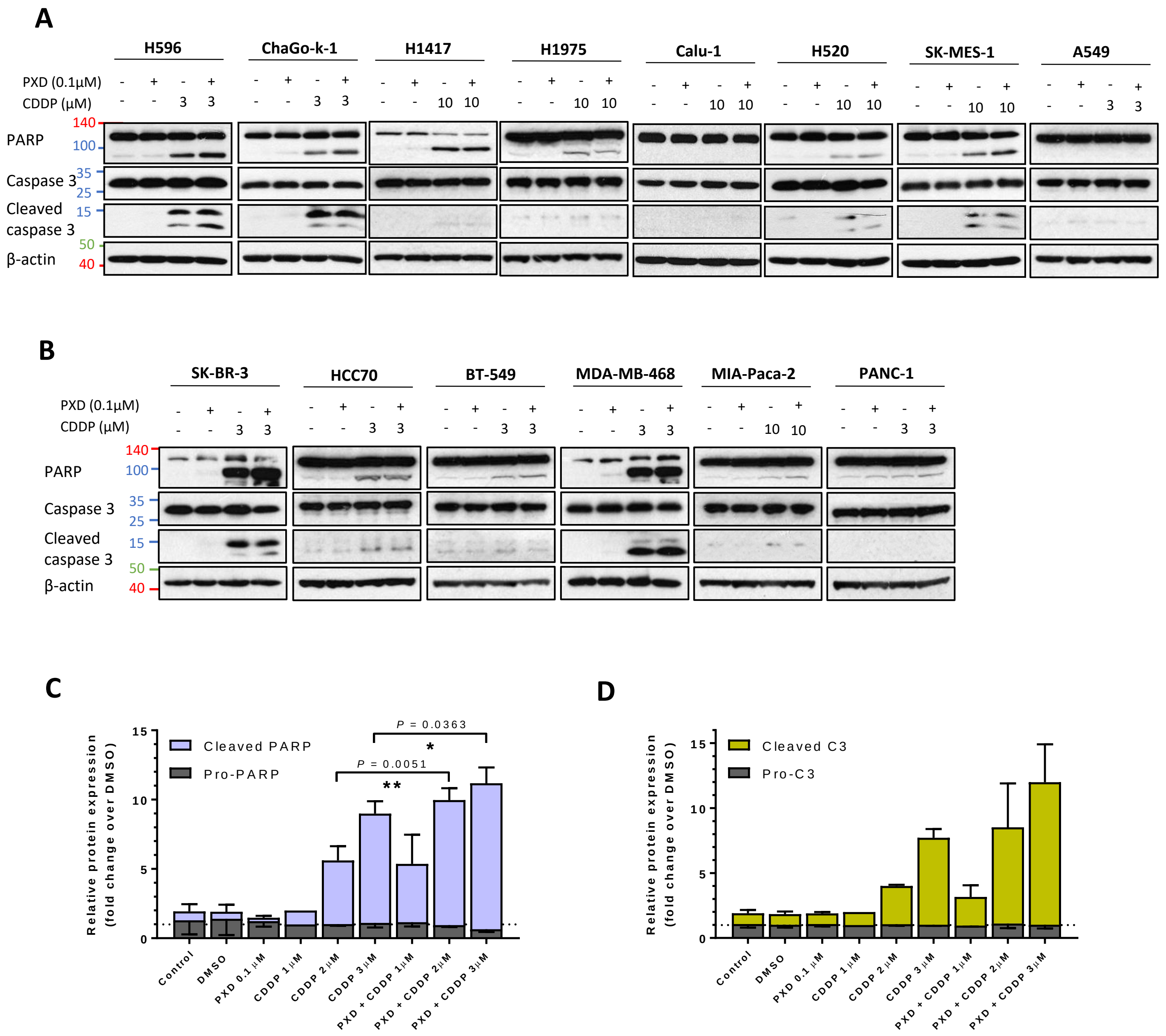

Supplementary Figure 3:

Synergistic cytotoxicity of cisplatin and belinostat in carcinoma cells with hotspot mutp53 status.

(A-B) Western blot measuring the changes in PARP and caspase-3 in lung cancer cells [H596 (G245C), ChaGo-k-1 (C275F), H1417 (R175L), H1975 (R273H), Calu-1 (null), H520 (W146*), SK-MES-1 (E298*) and A549 (wt)] (A); breast cancer cells [BT-549 (R249S), HCC7 (R248Q), MDA-MB-468 (R273H) and SK-BR-3 (R175H)], and pancreatic cancer cells [MIA-Paca-2 (R248W) and PANC-1 (R273H)] (B) after 48 hours treatment with belinostat (PXD101; 0.1 µM) and cisplatin (CDDP; 3 or 10 µM). β-actin shown as loading control (*n* = 3 independent experiments).

(C-D) Densitometric quantification of pro/cleaved PARP (C) and pro/cleaved caspase 3 (D) respectively for blots in Figure 3D. Relative fold change is normalized to β-actin, relative to single cisplatin treatment (1 µM). Data are represented as mean ± SD (*n* = 3 independent experiments). Two tailed Student's t-test; $^*P < 0.05$, $^{**}P < 0.01$.

Supplementary Figure 4

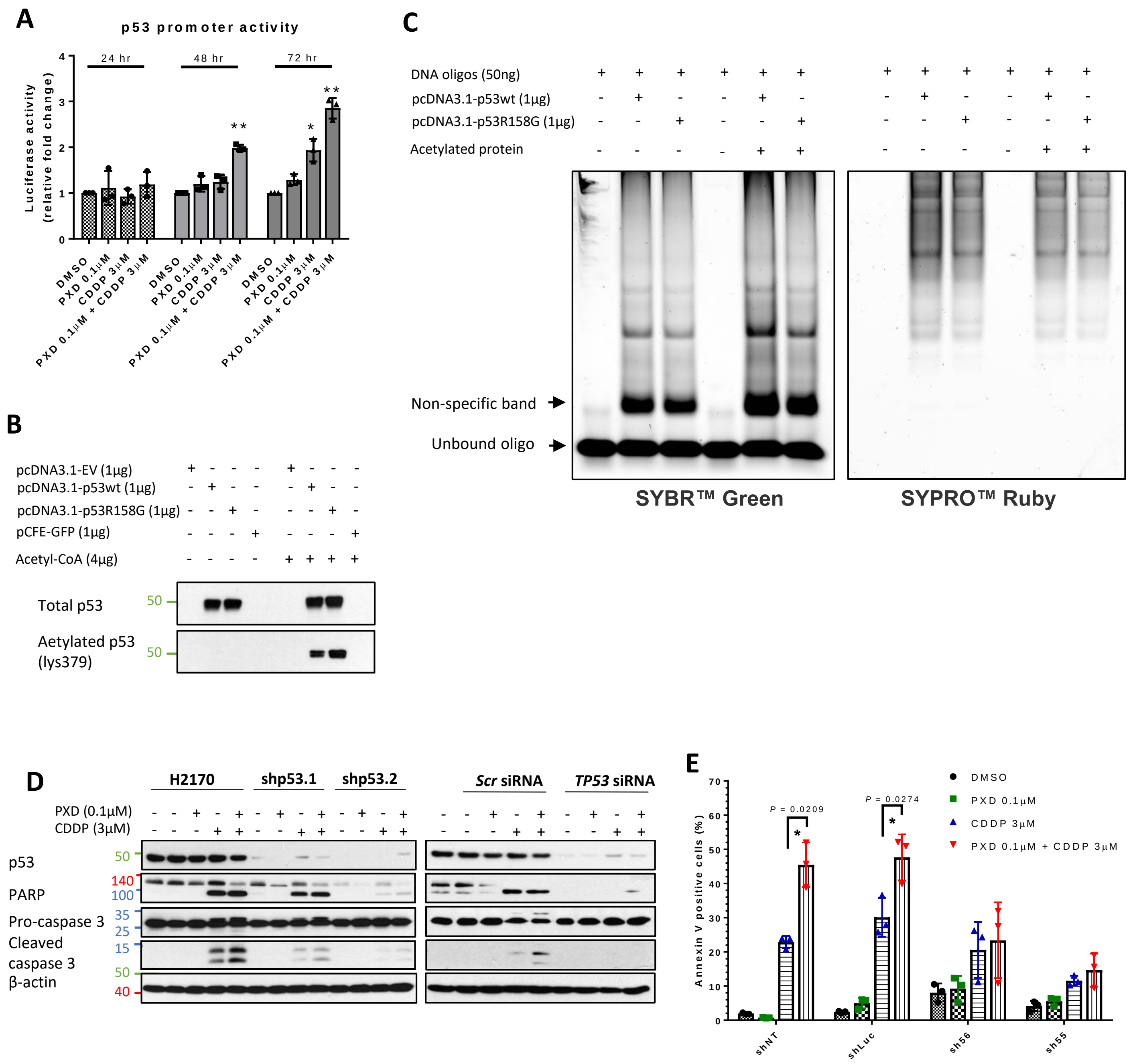

Supplementary Figure 4

DNA binding and transactivation of mutp53 is required for induction of apoptosis.

(A) Reporter assay measuring p53 promoter activity in H2170 cells after treatment with belinostat (PXD101; 0.1 μM) and cisplatin (CDDP; 3 μM) for 24, 48 and 72 hours. Data are represented as mean ± SD (n = 3 independent experiments). Two tailed Student's t-test; *$P < 0.05$, **$P < 0.01$.

(B-C) Electrophoretic mobility shift assay (EMSA) was performed using proteins expressed with *in vitro* translation. WT and Arg^{158} p53 were expressed from their respective plasmids, and protein acetylation was performed using the HAT domain of p300/HAT and acetyl-CoA. Western blot detects the expressed and modified proteins (B). The expressed proteins were incubated with 50ng of DNA oligos, and separated on a 6% non-denaturing TBE gel. SYBR Green (left) and SYPRO Ruby (right) were used for detection of nucleic acid and protein respectively (n = 2 independent experiments).

(D) Western blot indicates changes in p53, PARP and caspase-3 in shRNA-mediated (sh*p53*.1, sh*p53*.2) (left) or siRNA-mediated (scrambled and *TP53 siRNA*) (right) knockdown cells. 50 nM of siRNA was used per transfection. β-actin shown as loading control (n = 3 independent experiments).

(E) Extent of apoptosis was quantified with Annexin V staining in vector control (shNT and shLuc) or p53 knockdown (sh*p53*.1, sh*p53*.2) stable cells. Data are represented as mean ± SD (n = 3 independent experiments). Two tailed Student's t-test; *$P < 0.05$.

Supplementary Figure 5

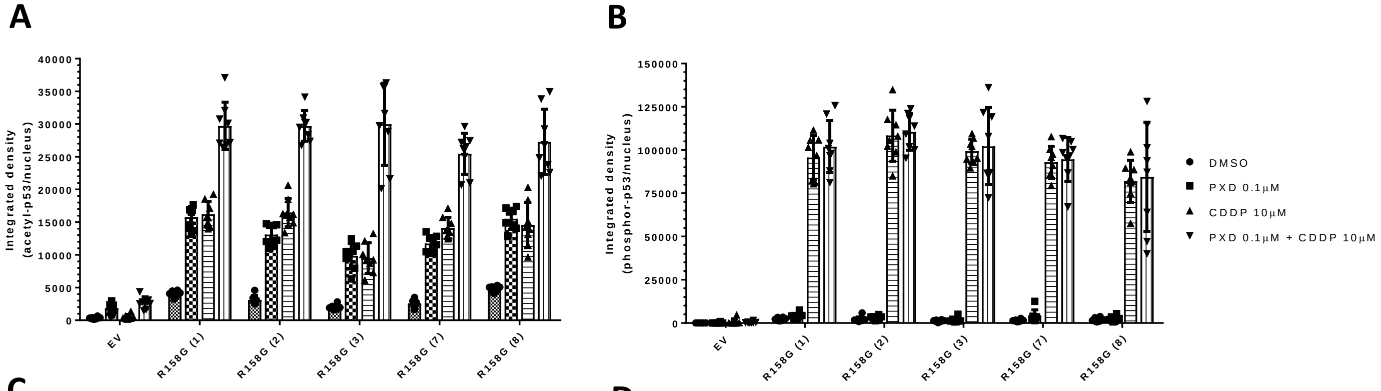

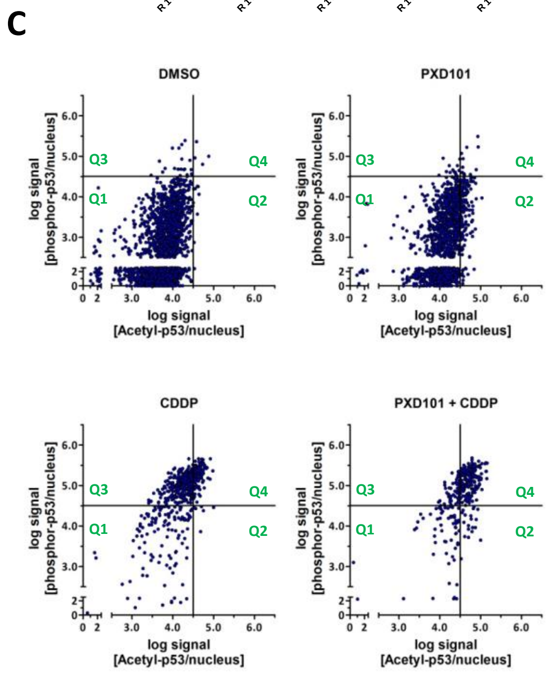

D

		% cell				
		$p53^{R158G(1)}$	$p53^{R158G(2)}$	$p53^{R158G(3)}$	$p53^{R158G(7)}$	$p53^{R158G(8)}$
DMSO	Q1	95.17	95.99	98.45	96.72	96.30
	Q2	2.17	1.42	0.44	1.36	1.98
	Q3	1.84	2.27	0.92	1.31	1.09
	Q4	0.81	0.33	0.19	0.61	0.64
PXD 0.1μM	Q1	71.51	79.28	88.78	83.42	74.22
	Q2	23.80	17.13	9.56	10.86	22.03
	Q3	0.67	1.17	0.86	2.40	1.18
	Q4	4.02	2.42	0.80	3.32	2.57
CDDP 10μM	Q1	22.79	22.52	22.92	22.94	27.45
	Q2	2.55	0.58	1.14	0.80	3.19
	Q3	34.69	38.51	54.36	42.84	35.53
	Q4	39.97	38.39	21.59	33.42	33.83
PXD 0.1μM CDDP 10μM	Q1	21.36	18.38	19.29	21.49	22.62
	Q2	5.99	4.37	6.11	3.44	11.31
	Q3	13.77	17.48	17.36	21.49	15.74
	Q4	58.88	59.77	57.23	53.58	50.33

Supplementary Figure 5:
Belinostat and cisplatin treatment differentially induce post-translational modifications of nuclear p53.
(A-B) High content analyses were performed for signal intensity of acetyl- (A) and p-p53 (B) in cell nucleus (immunofluorescence staining in Figure 4J). 8 fields were taken in each treatment group. Integrated density for each nucleus was determined by ImageJ. Data are represented as mean of each field ± SD in a representative experiment (*n* = 3 independent experiments).
(C) Scatter plots of individual nuclei for $p53^{R158G}$ are shown (X: log signal for acetyl-p53; Y: log signal for p-p53). Each scatter plot consists of > 400 nuclei. Gating was determined from vehicle control, upward shift indicates increase in phosphorylated p53, rightward shift indicates acetylated-p53.
(D) Tabulation of percentage of cell distribution for each $p53^{R158G}$ clones is displayed (Q1: p-p53 negative, acetyl-p53 negative; Q2: p-p53 negative, acetyl-p53 positive; Q3: p-p53 positive, acetyl-p53 negative; and Q4: p-p53 positive, acetyl-p53 positive), expressed as mean ± SD (*n* = 3 independent experiments).

Supplementary Figure 6

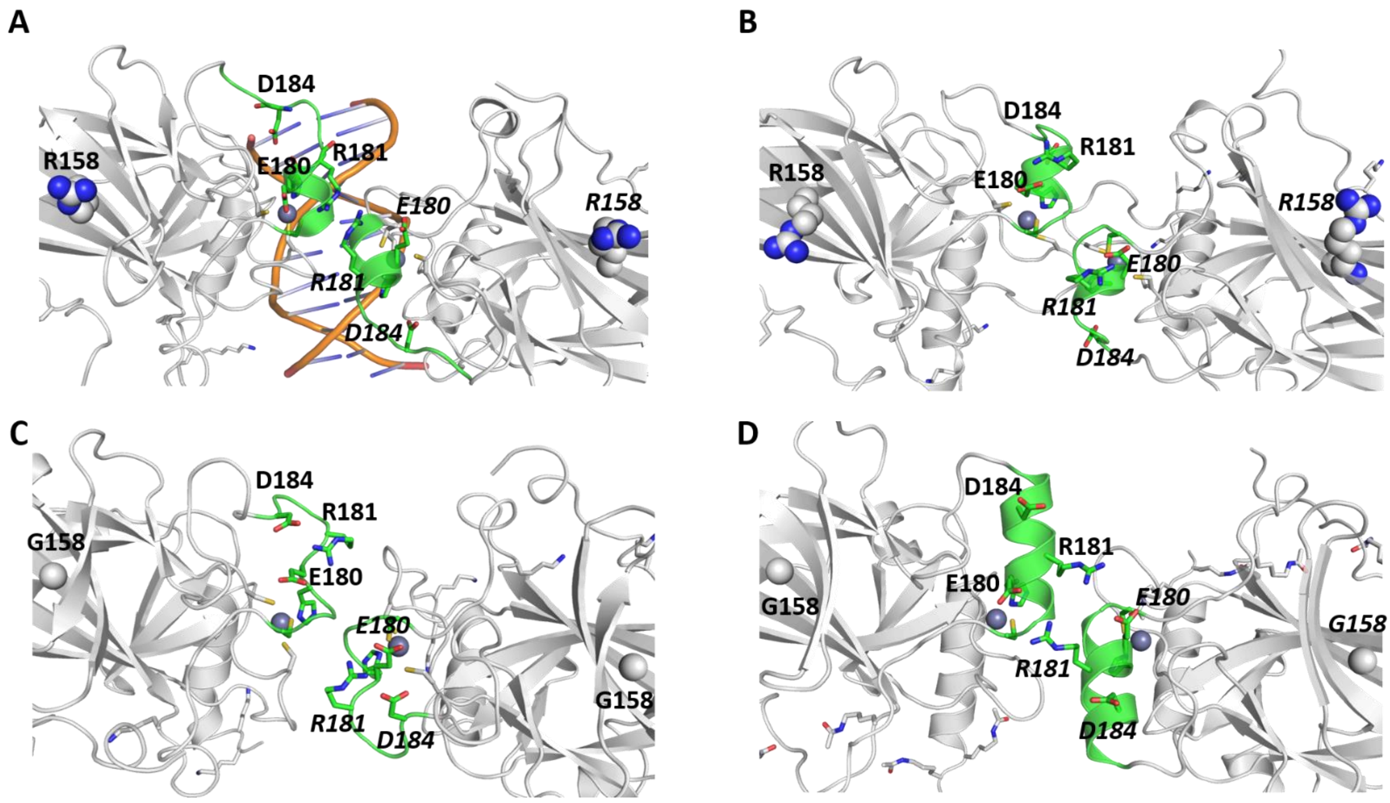

Supplementary Figure 6:
Structural simulation predicts that acetylation of the lysine residues could enhance DNA binding of mutp53^{R158G} molecule.
(A) The panel show the crystal structure of the dimer of the DNA binding domain (DBD) of p53 bound to a fragment of DNA (PDB id 2AHI) with key residues shown. The gray colored cartoon depicts the DBD while the orange cartoon is the DNA backbone with sticks representing the nucelobases in A; the black sphere represents a Zinc ion which is required for stabilizing the DBD; the R158 sidechain is shown as grey sphere (carbon atoms) and blue spheres (nitrogen atoms); the Lysine residues are shown as gray sticks with the terminal nitrogen colored in blue while upon acetylation, the sidechain oxygen is shown as red; the other sidechains shown are colored as: carbon – grey, oxygen – red, nitrogen – blue; the dimerization helix is shown as green.
(B-D) The panels show the conformations taken from the Molecular Dynamics simulations of the DBD in its WT (B), mutant R158G (C) and acetylated-Lys-R158G (D) forms.

Supplementary Figure 7

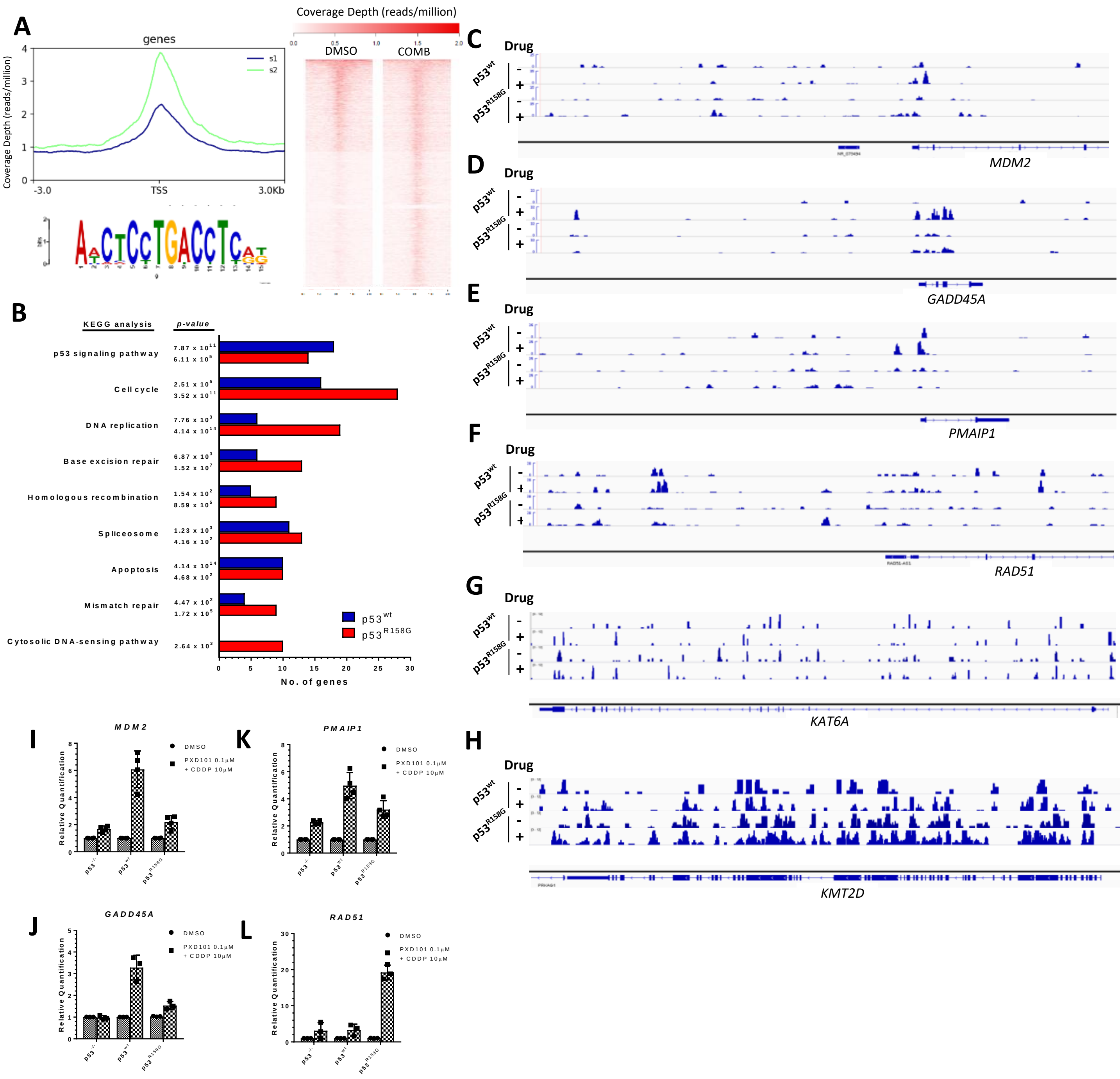

Supplementary Figure 7:

Comparative analyses of the genome-wide binding and transcriptomic regulations reveal differential expression patterns of wtp53 and $p53^{R158G}$.

(A) $p53^{wt}$-binding loci identified by p53 ChIP-seq analysis on Calu-1 cells treated with vehicle or belinostat/cisplatin combination for 24 hours (n = 1). Meta-peak analysis showing distribution of $p53^{wt}$-binding sites across 3,000 bp from the TSS of the nearest downstream gene (Left, top). Density heatmap of the wtp53-binding sites (±3,000 bp from TSS) examined by ChIP-seq (Right). Canonical wild-type p53 consensus motif was identified by MEME/TomTom from the TSS-proximal ChIP-Seq peaks (bottom left).

(B) KEGG enrichment (number of genes and statistics) of wtp53 and mutp53-induced genes upon drug treatment from AmpliSeq analysis.

(C-H) Integrative Genomics Viewer display of mutp53 occupancy over promoter region of *MDM2* (C), *GADD45A* (D), *PMAIP1* (E), *RAD51* (F), *KAT6A* (G), *KMT2D* (H) genes in vehicle- or drug-treated cells ($p53^{wt}$, $p53^{R158G}$).

(I-L) RT-qPCR quantification of *MDM2* (I), *GADD45A* (J), *PMAIP1* (*Noxa*) (K), *RAD51* (L) genes in Calu-1 ($p53^{-/-}$, $p53^{wt}$, $p53^{R158G}$) cells 48 hours after vehicle or drug treatment. Data are represented as average relative quantification ± SD (n = 3 independent experiments).

Supplementary Figure 8

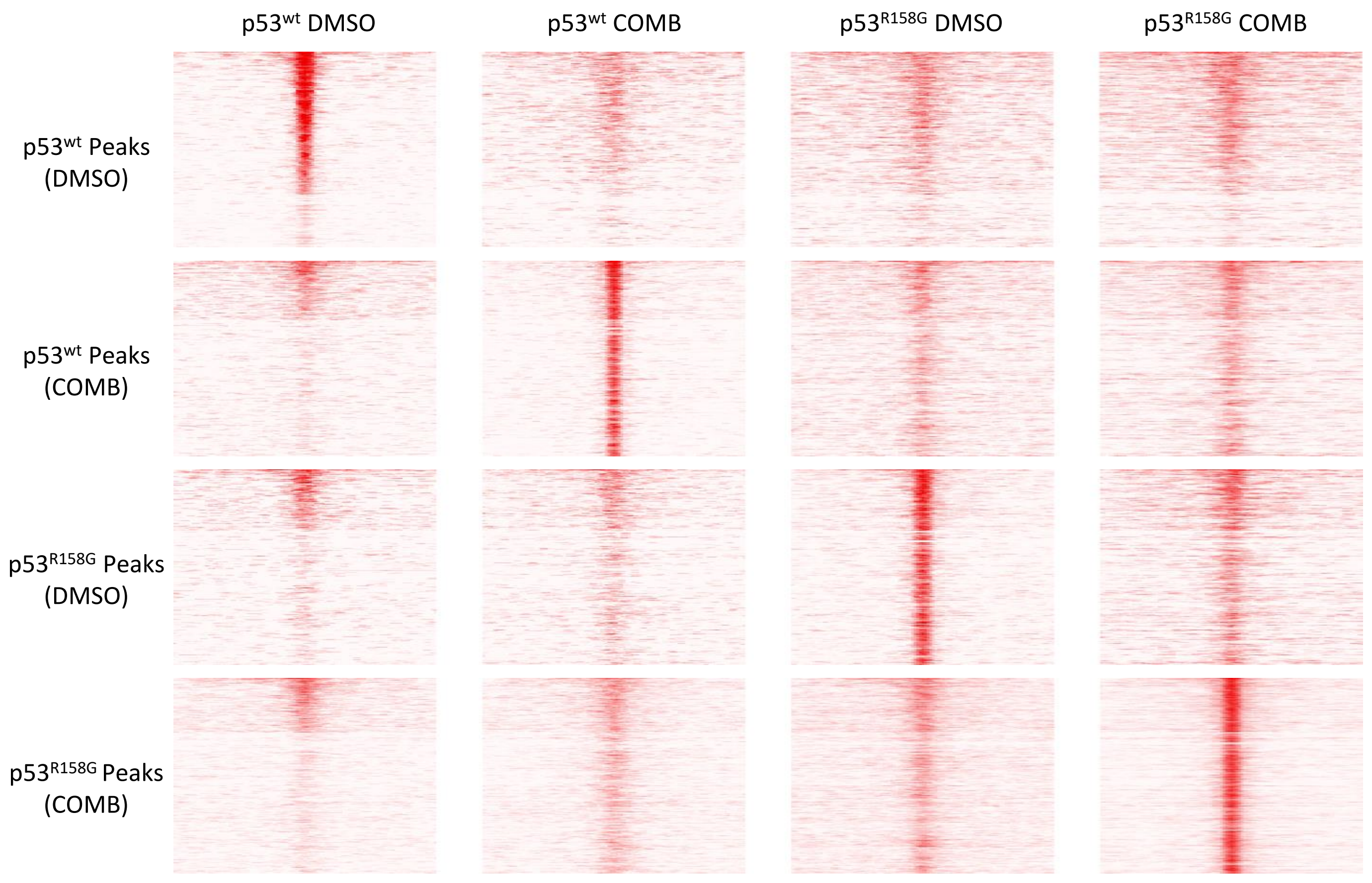

Supplementary Figure 8:
Distinctive genome-wide binding patterns of p53^{R158G} from that of wild-type p53.
Heatmaps showing the enrichment of p53 ChIP-Seq peaks (± 3,500 bp from peak center) identified from p53^{wt} or p53^{R158G} cell line in vehicle- or belinostat/cisplatin-treated conditions (row), over the peaks of p53 occupancy in all four conditions (column).

Supplementary Figure 9

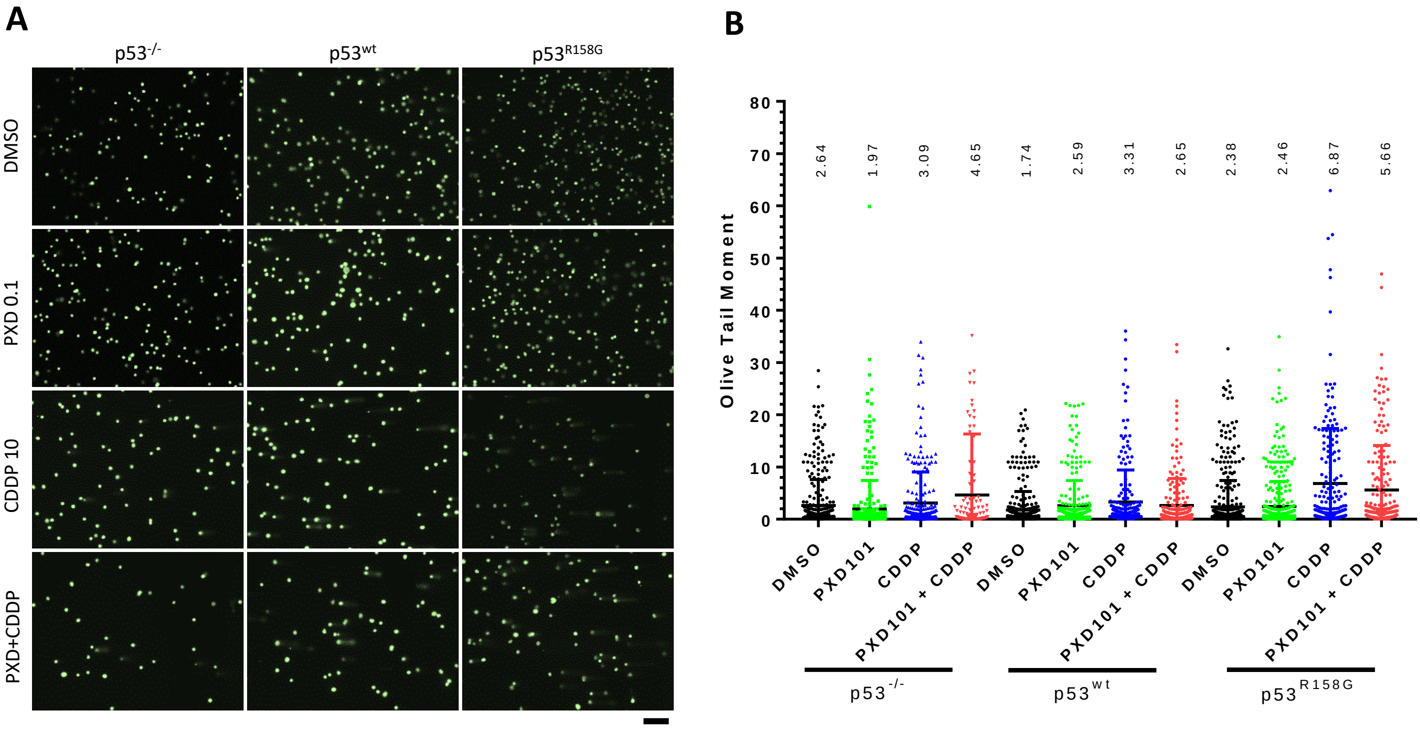

Supplementary Figure 9:

Comet tail analysis of DNA damage in wtp53 and p53^{R158G} cells after exposure to belinostat and/or cisplatin.

(A) Comet assay measuring the extent of DNA damage 48 hours after treatment with belinostat (PXD101; 0.1 μM) and cisplatin (CDDP; 10 μM) in Calu-1 (p53$^{-/-}$, p53^{wt}, p53^{R158G}) cells. Immunofluorescence images was visualized and captured to determine the amount of DNA damage present in individual nuclei. At least four independent fields were taken for each condition with a minimum of 100 nuclei per group (*n* = 3 independent experiments). Representative confocal images are shown at 20× magnification. Scale bar, 50 μm.

(B) The relative length and intensity of Vista Green-stained DNA tails to heads (measured as olive tail moment, values indicated at the top) in each group. Individual olive tail moment of all cells in each group is plotted and presented as mean ± SD in one representative experiment (*n* = 3 independent experiments).

Supplementary Figure 10

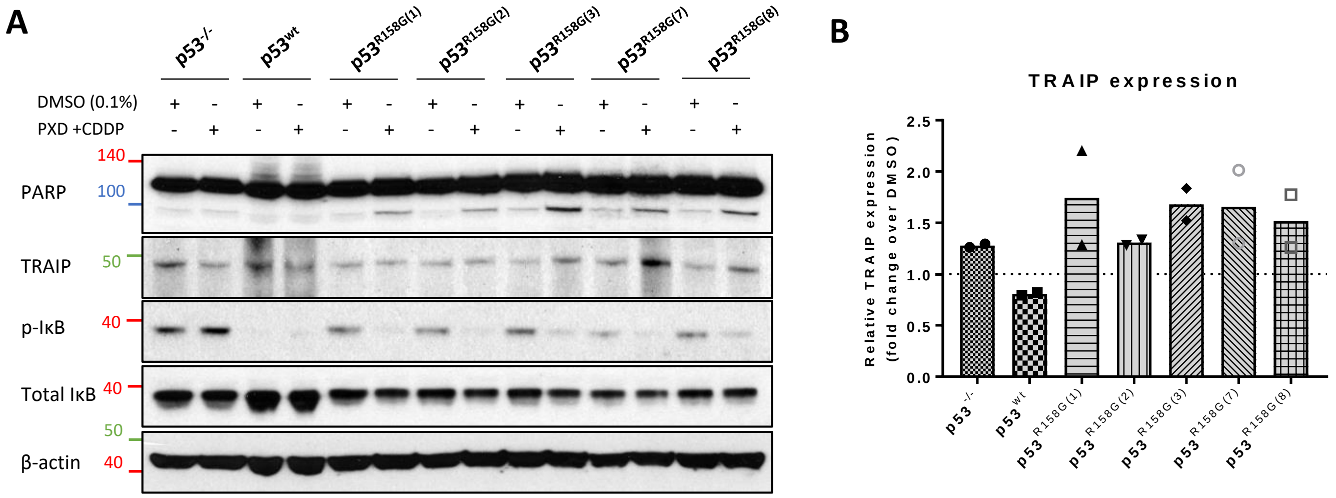

Supplementary Figure 10:
Belinostat and cisplatin co-treatment induced TRAIP expression and p-IκB suppression in p53^{R158G} cells.
(A-B) Immunoblotting was performed to evaluate the effects of belinostat/cisplatin on the indicated targets in various p53 clones 48 hours post-treatment (A). β-actin shown as loading control. Densitometric quantification of TRAIP blots (B), normalized to β-actin, is tabulated (*n* = 2 independent experiments).

Supplementary Figure 11

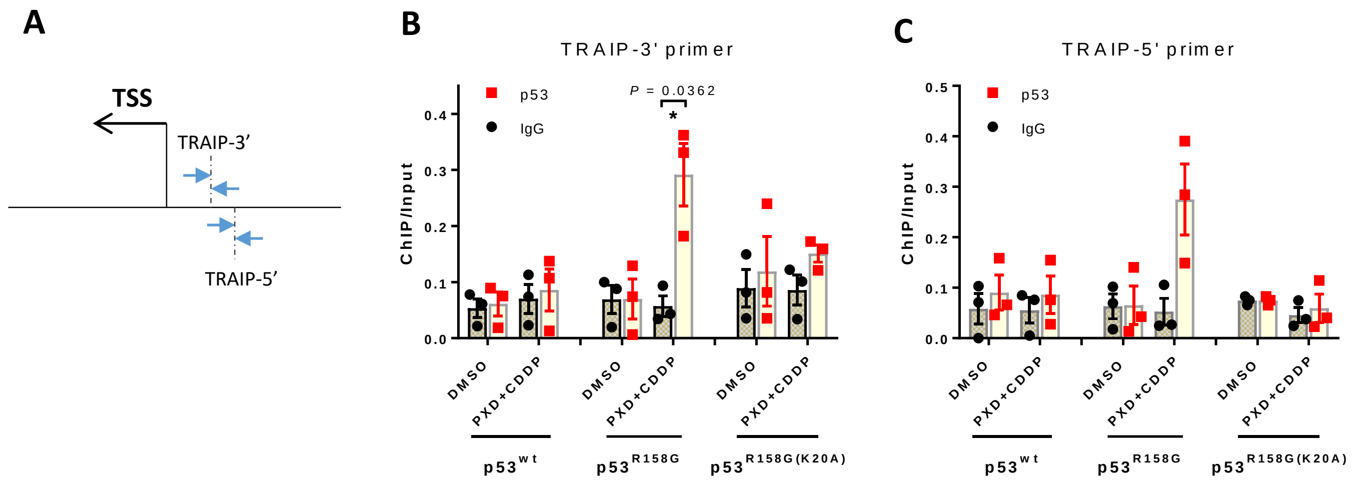

Supplementary Figure 11:
ChIP-qPCR validation of mutp53 binding at *TRAIP* gene.
(A) Schematic presentation of amplicon locations for qPCR validation of ChIP-Seq target gene. Primers (TRAIP-3' and TRAIP-5') flanking promoter region of *TRAIP* gene.
(B-C) qPCR showing p53 ($p53^{wt}$, $p53^{R158G}$, $p53^{R158G(K20A)}$) or IgG enrichment over *TRAIP* promoter region in Calu-1 cells. Primers were designed to enrich the 3' (B) or 5' (C) regions of the promoter. Signals obtained from the ChIP are compared against the input chromatin amount (ChIP/Input), and presented as mean ± SEM (n = 3 independent experiments). Two tailed Student's t-test, $^*P < 0.05$.

Supplementary Figure 12

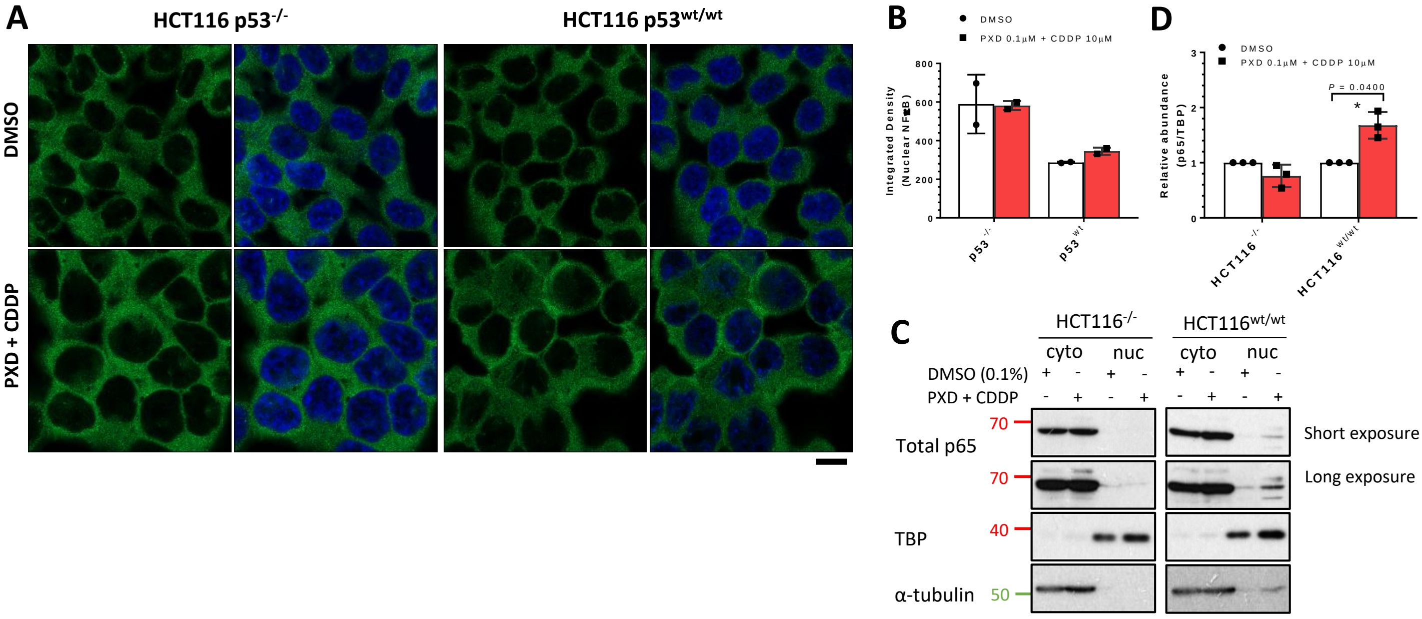

Supplementary Figure 12:

p53 status is a key determinant of NFκB signaling in HCT116 cells when treated with belinostat/cisplatin.

(A-B) Immunofluorescence staining was performed to determine the localization of p65 (Alexa Fluor-488) in HCT116 ($p53^{-/-}$ and $p53^{wt/wt}$) cells in vehicle- or drug-treated cells 48 hours post-treatment. At least five independent fields were taken for each condition with a minimum of 50 nuclei per group. Representative confocal images are shown at 63x magnification (A). Scale bar, 10 μm. Merged images are displayed with blue indicates DAPI, green indicates p65. Integrated density quantifying nuclear NFκB (p65) upon treatment in both HCT116 cells (B). Data are presented as mean ± SD (n = 2 independent experiments).

(C-D) Distribution of p65 (in cytoplasm or nucleus) was determined by Western blot (C) after nuclear-cytosolic fractionation. TATA-box binding protein (TBP) (nuclear) and α-tubulin (cytoplasmic) were used as loading controls. Nuclear p65 signal was quantified with densitometry after normalizing to TBP (D). Data are presented as mean ± SD (n = 3 independent experiments). Two tailed Student's t-test; *P < 0.05.

Supplementary Figure 13

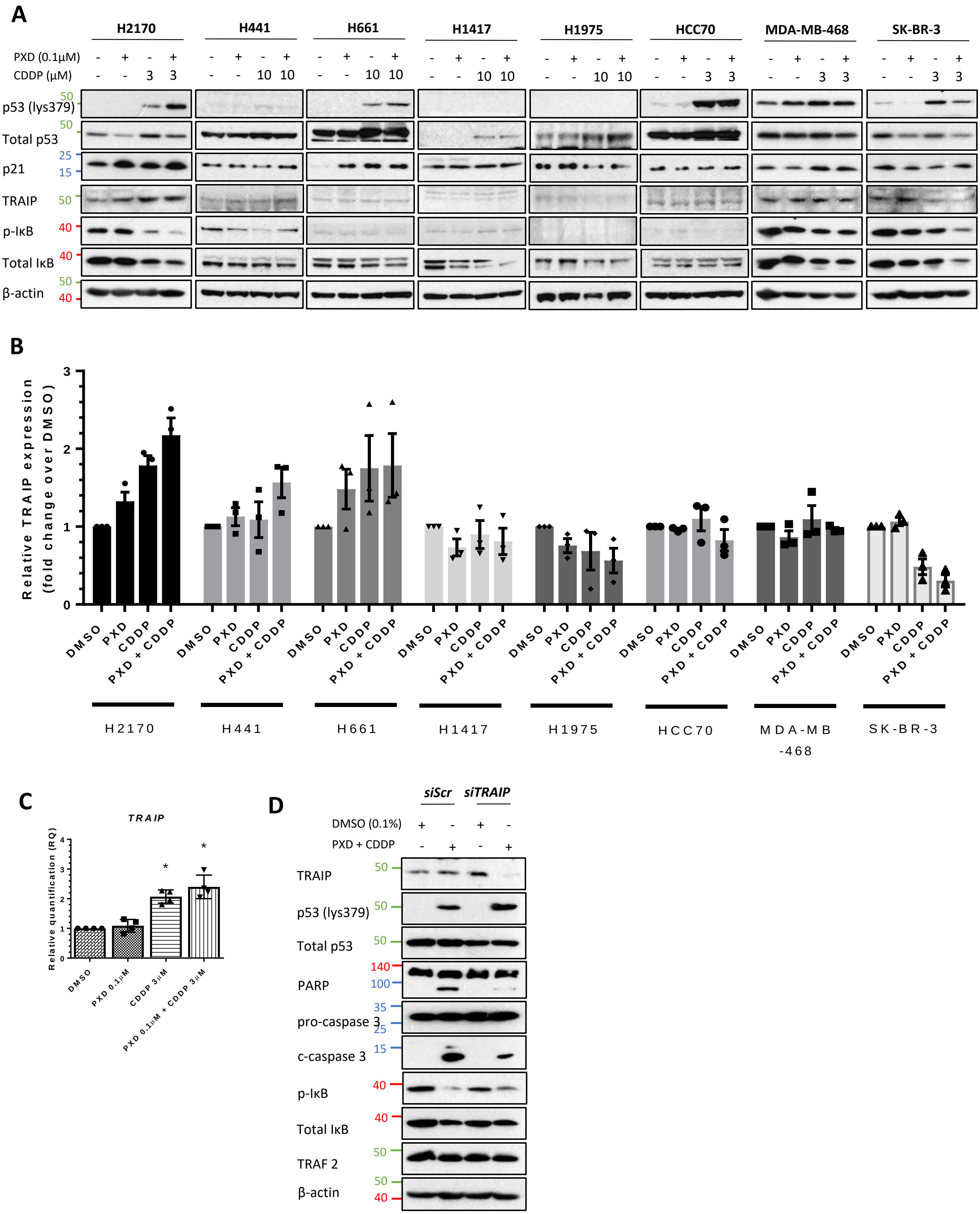

Supplementary Figure 13:
TRAIP activation is observed in codon 158 mutp53 cells and is critical for the induction of apoptosis.
(A-B) Western blot comparing expressions of the indicated targets among H2170 (R158G), H441 (R158L), H661 (R158L), H1417 (R175L), H1975 (R273H), HCC70 (R248Q), MDA-MB-468 (R273H) and SK-BR-3 (R175H) in response to 48 hours vehicle or drug treatment (A). β-actin shown as loading control. Densitometric quantification of TRAIP blots (B), normalized to β-actin, is tabulated + SEM (*n* = 3 independent experiment).
(C) RT-qPCR quantification of *TRAIP* gene in H2170 cells 48 hours after vehicle or drug treatment. Data are presented as average relative quantification ± SD (*n* = 3 independent experiemnt). Two tailed Student's t-test; *$P < 0.05$.
(D) Western blot evaluating effects of *TRAIP* knockdown upon treatment in H2170 cells (*n* = 3 independent experiments). 50 nM of siRNA was used per transfection. β-actin shown as loading control.

Supplementary Figure 14

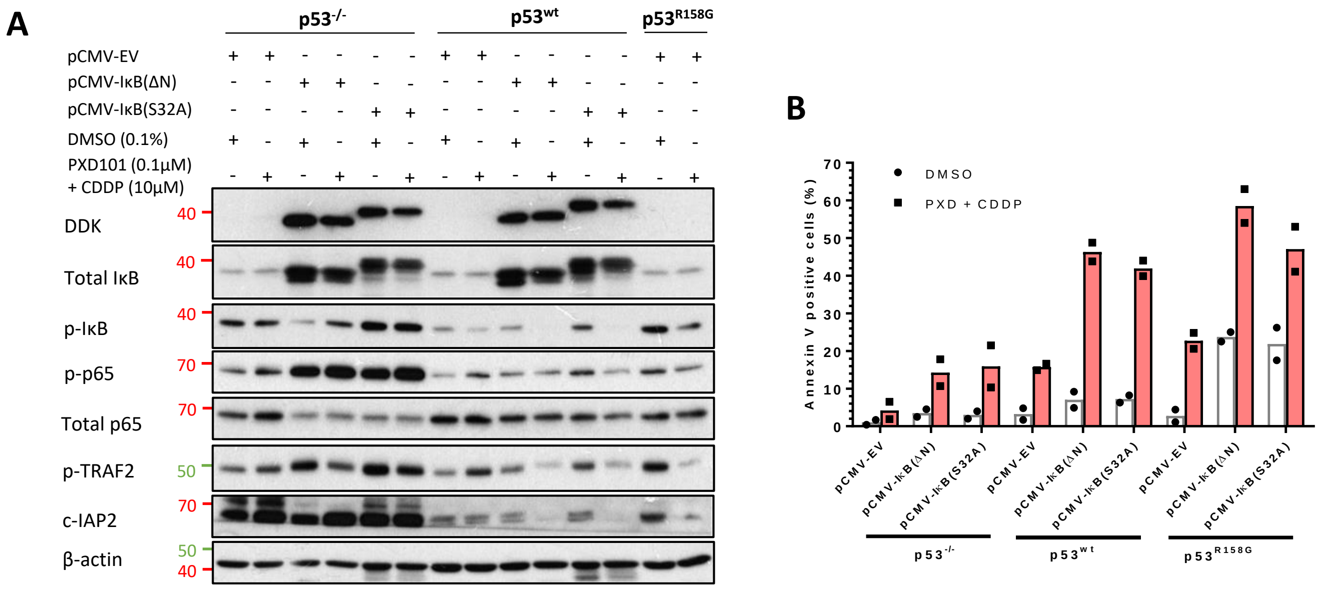

Supplementary Figure 14:
Forced suppression of NFκB signaling with dominant negative IκB expression enhances cytotoxicity in both wtp53 and p53^{R158G} cells.
(A) Western blot showing effects of dominant negative IκB (pCMV-IκB(ΔN), pCMV-IκB(S32A)) on the indicated proteins in Calu-1 (p53$^{-/-}$, p53^{wt}, p53^{R158G}) cells 48 hours after vehicle or drug treatment (*n* = 3 independent experiments). β-actin shown as loading control.
(B) Quantification of apoptotic cell population with Annexin V staining in vector control or IκB-overexpressed cells 48 hours post treatment. Data are represented as mean ± SD (*n* = 2 independent experiments).

Supplementary Figure 15

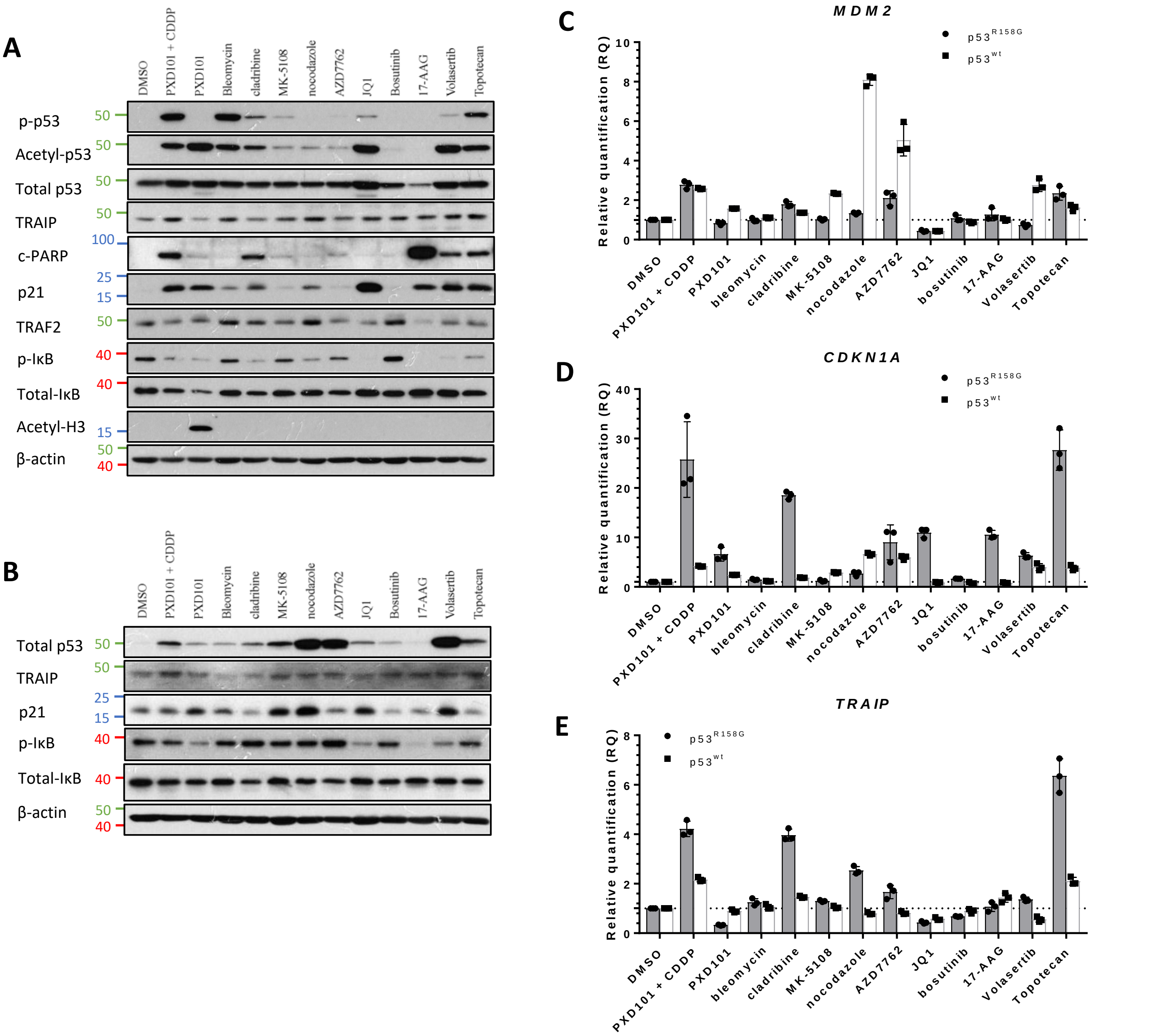

Supplementary Figure 15:

Compounds demonstrating selectivity towards $p53^{R158G}$ cells are mostly potent acetylating agent of mutp53.

(A-B) Downstream effects of selected compounds (agents with higher sensitivity towards $p53^{R158G}$ cells, identified in Figure 2A) were evaluated in Calu-1 isogenic clones after 48 hours treatment. Western blots comparing regulation of the indicated proteins in $p53^{R158G}$ (A) and $p53^{wt}$ (B) cells. β-actin shown as loading control (n = 3 independent experiments).

(C-E) RT-qPCR quantification of *MDM2* (C), *CDKN1A* (D) and *TRAIP* (E) genes in $p53^{R158G}$ and $p53^{wt}$ cells 48 hours after vehicle or drug treatment. Data are presented as average relative quantification ± SD (n = 3 independent experiments).

Supplementary Figure 16

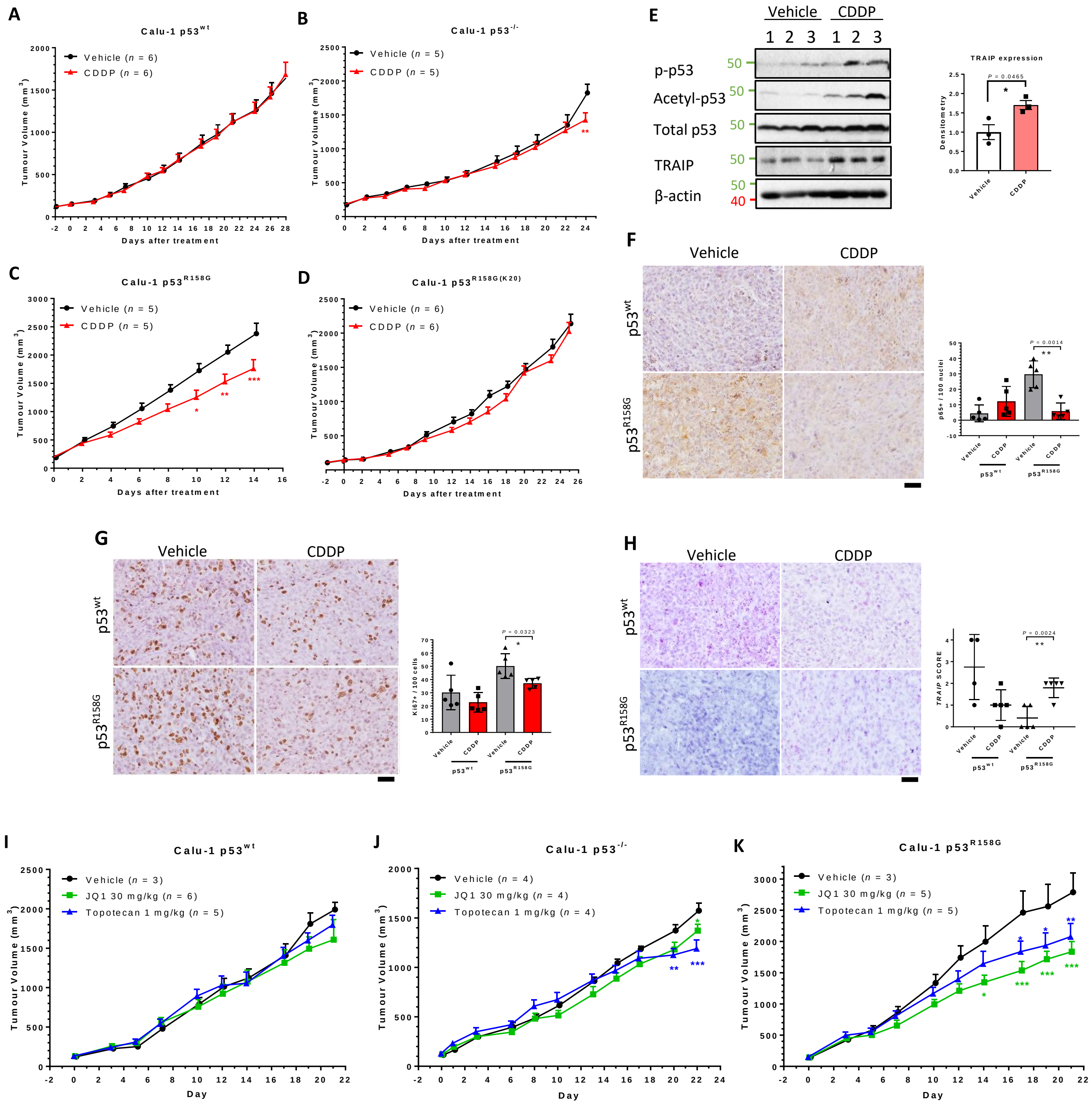

Supplementary Figure 16:
Cisplatin, topotecan and JQ1 selectively reduce growth of mutp53^{R158G} tumors.
(A-D) Growth curve analysis of Calu-1 p53^{wt} (A), p53$^{-/-}$ (B), p53^{R158G} (C) and p53$^{R158G(k20A)}$ (D) in xenografts treated with vehicle or cisplatin (CDDP; 4 mg/kg). Tumor sizes are presented as mean ± SEM. Two-way ANOVA with Bonferroni correction; *P < 0.05; **P < 0.01; ***P < 0.001.
(E) Western blots demonstrating changes of the indicated proteins in tumors of respective treatment (n = 3 independent tumours). β-actin shown as loading control. Densitometric quantification of TRAIP expression was tabulated on the right. Relative fold change is normalized to β-actin, relative to vehicle control tumors and presented at mean ± SEM. Two tailed Student's t-test; *P < 0.05.
(F-G) Immunohistochemistry staining analyses of intracellular expressions of p65 and Ki67 in respective tumors. Representative images at 20× showing p65 staining (F) and Ki67 staining (G). Scale bar, 50 μm. Quantification of positively-stained cells (%) in p53^{wt} and p53^{R158G} respectively was tabulated on the right. Data are represented as percentage of positive cells ± SD (n = 5 independent tumours). Two tailed Student's t-test; for xenograft models, *P < 0.05, **P < 0.01.
(H) RNA *in situ* hybridisation (RNAscope) showing *TRAIP* expression in respective tumors. Representative images showing *TRAIP* mRNA signal. Semi-quantitative scoring (0-4) of *TRAIP* mRNA signal (dots/cell) in p53^{wt} and p53^{R158G} respectively was tabulated on the right. Data are represented as scattered dot plot ± SD (n = 5 independent tumours). Scale bar, 50 μm. Two tailed Student's t-test; for xenograft models, **P < 0.01.
(I-K) Growth curve analysis of Calu-1 p53^{wt} (I), p53$^{-/-}$ (J) and p53^{R158G} (K) in xenografts treated with vehicle, JQ1 (30 mg/kg) or topotecan (1 mg/kg). Tumor sizes are presented as mean ± SEM. Two-way ANOVA with Bonferroni correction; *P < 0.05; **P < 0.01; ***P < 0.001.

Supplementary Table 1

Disease	Total samples	Total Number of Sample with Mutated *TP53*	Total number of sample with R158 mutation	Total number of sample with R158 mutation (%)
**Lung Squamous Cell Carcinoma**	**178**	**146**	**8**	**4.49**
Uterine Corpus Endometrial Carcinoma	33	10	1	3.03
**Lung adenocarcinoma**	**564**	**295**	**8**	**1.42**
Esophageal carcinoma	186	153	2	1.08
Liver Hepatocellular carcinoma	199	63	2	1.01
Bladder urothelial carcinoma	131	64	1	0.76
Head and Neck squamous cell carcinoma	280	205	2	0.71
Stomach Adenocarcinoma	290	138	2	0.69
Glioblastoma multiforme	577	230	3	0.52
Sarcoma	248	85	1	0.40
Breast invasive carcinoma	994	309	1	0.10

Supplementary Table 1:
Prevalence and distribution of TP53 codon 158 mutation in various carcinomas according to The Cancer Genome Atlas (TCGA) database. Cases of lung carcinomas were highlighted in red.

Supplementary Table 2

TP53 hotspot	LUAD cases with *TP53* Mut (*N* = 295 out of 564)		LSCC cases with *TP53* Mut (*N* = 146 out of 178)		Lung carcinomas with *TP53* Mut (*N* = 2397 out of 6793)		Non-lung carcinomas with *TP53* Mut (*N* = 1956 out of 6051)	
	Total #	% cases	Total #	% cases	Total #	% cases	Total #	% cases
158	8	2.71	**8**	**5.48**	31	1.29	15	0.77
175	3	1.02	3	2.05	97	4.05	91	4.65
245	8	2.71	5	3.42	54	2.25	41	2.10
248	5	1.69	4	2.74	**126**	**5.26**	**117**	**5.98**
249	**10**	**3.39**	3	2.05	31	1.29	18	0.92
273	9	3.05	6	4.11	**123**	**5.13**	**108**	**5.52**
282	3	1.02	2	1.37	41	1.71	36	1.84

Supplementary Table 2:
Distribution of various TP53 hotspot mutations (codon 158, 175, 245, 248, 249, 273, 282) in lung adenocarcinoma (LUAD) and lung squamous cell carcinoma (LUSC) according to The Cancer Genome Atlas (TCGA) database. Prevalent cases were highlighted in red.

Supplementary Table 3

Compound	p53^{R158G}	p53$^{-/-}$	p53^{wt}	p53^{R158G}	p53$^{-/-}$	p53^{wt}	Target
	0.1 µM			1 µM			
AC:Docetaxel (Taxotere)	59.5	57.3	42.2	74.9	68.4	44.5	Microtubule Associated
AC:Torin 2	38.9	47.8	24.1	64.3	61.9	41.2	mTOR
AC:Simvastatin (Zocor)	0.0	10.1	15.4	64.6	71.8	52.3	
AC:Romidepsin (FK228, Depsipeptide)	91.3	93.5	89.8	94.6	95.1	93.4	HDAC
AC:MLN2238	45.9	73.6	52.9	92.1	93.8	90.7	Proteasome
AC:Paclitaxel (Taxol)	44.5	59.0	38.5	71.9	64.5	48.5	Microtubule Associated
AC:Flavopiridol (Alvocidib)	26.7	25.6	29.8	73.8	88.6	60.3	CDK
AC:Epothilone A	73.7	72.1	55.1	79.4	75.1	47.4	Microtubule Associated
AC:GSK2126458	1.3	0.0	11.2	28.6	54.5	43.4	PI3K, mTOR
AC:AZD7762	8.7	0.0	0.0	69.6	45.3	44.7	Chk
AC:Nocodazole	0.0	10.6	6.5	55.3	46.3	39.2	Microtubule Associated
AC:MLN9708	29.8	49.9	35.5	91.6	94.4	92.1	Proteasome
AC:GSK923295	21.2	56.5	23.9	42.1	64.2	39.9	Kinesin
AC:Bortezomib (Velcade)	85.7	92.0	89.0	93.9	93.7	91.2	Proteasome
AC:Elesclomol	75.6	94.6	66.9	94.4	94.6	93.8	HSP
AC:Topotecan HCl	43.7	26.3	11.8	58.3	21.8	15.9	Topoisomerase
AC:Floxuridine (Fludara)	37.2	21.4	18.7	68.0	50.5	24.3	DNA/RNA Synthesis
AC:Gemcitabine (Gemzar)	53.5	65.9	38.3	61.0	64.8	44.9	
AC:GSK461364	42.8	42.9	49.8	65.8	54.3	43.4	PLK
AC:PF-3758309	11.0	49.4	17.8	42.1	61.6	26.7	PAK
AC:KX2-391	36.8	64.5	27.8	69.5	76.0	38.5	Src
AC:Mitoxantrone	34.6	37.7	10.0	71.1	70.0	73.3	Topoisomerase
AC:Bexarotene	37.5	53.7	41.1	35.1	46.7	19.8	
AC:CYT997 (Lexibulin)	40.7	41.8	28.2	78.1	81.2	43.5	Microtubule Associated
AC:HSP990 (NVP-HSP990)	64.1	60.6	38.5	83.5	80.9	48.3	HSP
AC:Cladribine	0.0	16.6	0.0	51.5	38.3	25.9	DNA/RNA Synthesis
AC:SB939 (Pracinostat)	19.4	0.1	13.4	51.7	34.7	55.7	HDAC
AC:Triptolide	65.5	86.5	42.6	81.4	91.8	67.1	
AC:Ganetespib (STA-9090)	77.5	70.8	34.4	78.4	73.6	45.2	HSP
AC:BIIB021	11.3	2.6	0.0	70.1	60.1	45.3	HSP
AC:Trichostatin A (TSA)	11.9	0.0	1.4	87.9	83.3	55.3	HDAC
AC:AUY922 (NVP-AUY922)	77.5	74.0	29.3	77.9	72.3	43.4	HSP
AC:BI 2536	62.8	54.6	33.6	58.6	39.0	18.0	PLK
AC:Flavopiridol (Alvocidib) HCl	0.0	7.1	0.0	68.1	86.9	57.5	CDK
AC:Disulfiram (Antabuse)	0.0	0.0	0.0	41.3	70.9	55.6	
AC:Bosutinib (SKI-606)	16.9	0.0	4.8	52.7	49.8	13.9	Src
AC:GW3965 HCl	44.7	20.9	45.9	62.9	59.0	67.5	Liver X Receptor
AC:Deltarasin	0.0	13.0	8.9	22.7	50.6	47.2	PDE
AC:Ro3280	48.9	21.6	30.3	65.7	55.4	38.3	PLK
AC:Vinorelbine Tartrate	59.6	58.9	37.0	71.5	77.0	32.0	Microtubule Associated
AC:PFK15	5.8	4.5	10.0	19.8	72.6	8.1	
AC:VER-50589	37.5	9.4	0.0	82.9	73.5	29.9	HSP
AC:17-AAG (Tanespimycin)	0.0	5.8	0.0	56.2	48.2	0.0	HSP
AC:17-DMAG HCl (Alvespimycin)	78.6	68.7	21.8	77.0	68.2	31.4	HSP
AC:SNS-032 (BMS-387032)	12.1	6.5	0.0	39.6	57.6	44.7	CDK
AC:Pexmetinib (ARRY-614)	12.1	14.7	0.0	73.6	77.7	39.7	p38 MAPK
AC:INK 128 (MLN0128)	3.0	14.4	0.0	45.7	61.6	29.8	mTOR
AC:CH5138303	16.8	0.0	12.8	76.1	57.8	27.8	HSP
AC:Rigosertib (ON-01910)	43.0	40.7	1.1	79.2	68.9	38.7	PLK
AC:Epothilone B (EPO906)	70.2	68.6	46.8	74.1	72.0	52.0	Microtubule Associated
AC:CB-839	74.4	82.9	75.7	88.6	93.1	85.6	
AC:Pelitinib (EKB-569)	6.1	31.8	1.9	46.8	71.7	20.1	EGFR
AC:MI-2 (MALT1 inhibitor)	35.3	34.2	31.1	94.8	95.8	94.1	
AC:Obatoclax mesylate (GX15-070)	0.0	0.0	0.0	68.7	65.9	38.2	Bcl-2
AC:BI-847325	9.3	21.3	0.0	31.6	60.8	33.6	MEK
AC:JNJ-26481585	54.3	44.9	24.7	93.7	93.9	85.2	HDAC
AC:Ispinesib (SB-715992)	57.6	62.6	47.2	66.2	69.8	58.0	Kinesin
AC:ARQ 621	26.6	10.0	16.4	55.9	66.7	60.1	Kinesin

Compound	p53^{R158G}	p53$^{-/-}$	p53^{wt}	p53^{R158G}	p53$^{-/-}$	p53^{wt}	Target
	0.1 µM			1 µM			
AC:Tipifarnib (Zarnestra)	26.1	34.4	9.6	55.0	66.8	53.5	Farnesyltransferase, Ras
AC:PU-H71	18.6	39.6	7.9	61.0	56.8	20.8	HSP
AC:Entinostat (MS-275, SNDX-275)	29.1	23.2	3.4	56.8	28.8	51.8	HDAC
AC:Dasatinib (BMS-354825)	1.4	6.2	0.0	26.4	54.6	30.4	Src, Bcr-Abl, c-Kit
AC:4SC-202	16.4	4.4	16.9	47.2	54.7	23.8	HDAC
AC:BI6727 (Volasertib)	51.7	14.3	32.6	58.5	43.1	20.8	PLK
AC:JNK Inhibitor IX	0.0	5.0	7.2	57.6	58.2	30.8	JNK
AC:Picropodophyllin (PPP)	10.5	0.8	6.4	40.9	54.1	17.7	IGF-1R
AC:Plinabulin (NPI-2358)	39.6	31.7	7.6	73.1	69.8	41.3	VDA
AC:Bleomycin sulfate	34.9	21.4	6.9	59.5	43.7	10.7	DNA/RNA Synthesis
AC:Vincristine	59.5	63.7	28.1	79.2	80.6	35.8	Autophagy,Microtubule
AC:Voreloxin (SNS-595)	48.2	38.4	7.3	58.2	60.2	39.1	Topoisomerase
AC:Mitoxantrone HCl	35.2	12.8	0.0	66.5	65.3	67.6	
AC:LY3009120	23.8	9.1	9.8	50.7	70.3	21.7	Raf
AC:MK-1775	3.1	0.0	0.0	52.1	50.5	11.4	Wee1
AC:PIK-75	45.8	65.8	0.0	78.2	92.4	62.3	PI3K, DNA-PK
AC:Teniposide (Vumon)	43.9	11.2	0.0	69.6	55.1	11.9	
AC:Clofarabine	13.3	0.0	0.0	59.5	57.9	35.4	DNA/RNA Synthesis
SkEpi:JIB-04	0.0	9.3	15.9	67.3	62.7	87.8	Histone Demethylase
SkEpi:TAK-901	13.2	24.6	25.1	36.4	50.0	60.0	Aurora Kinase
SkEpi:LAQ824 (Dacinostat)	63.3	61.7	48.5	97.9	96.9	95.4	HDAC
SkEpi:M344	6.8	14.3	21.4	45.0	31.6	31.0	HDAC
SkEpi:(+)-JQ1	15.3	21.3	25.5	54.7	42.1	35.8	Epigenetic Reader Domain
SkEpi:ENMD-2076	17.7	11.7	23.3	33.0	46.1	41.5	Aurora Kinase,FLT3,VEGFR
SkEpi:4SC-202	0.0	7.9	17.1	68.9	61.5	60.8	HDAC
SkEpi:Pracinostat (SB939)	0.0	0.4	17.2	49.2	56.7	49.9	HDAC
SkEpi:TG101209	6.6	11.4	23.8	31.8	44.2	29.4	JAK,FLT3,c-RET
SkEpi:Hesperadin	14.4	25.2	46.6	62.2	70.6	70.2	Aurora Kinase
SkEpi:Trichostatin A (TSA)	16.4	24.8	32.1	97.5	94.0	89.1	HDAC
SkEpi:FG-4592	0.0	12.5	19.8	22.2	6.4	45.5	HIF
SkEpi:Quisinostat (JNJ-26481585)	94.5	86.3	81.2	98.3	98.2	98.1	HDAC
SkEpi:Romidepsin (FK228, Depsipeptide)	98.6	98.6	98.4	98.9	99.0	98.8	HDAC
SkEpi:CX-6258 HCl	27.9	25.1	21.1	52.7	41.9	41.3	Pim
SkEpi:CUDC-907	96.0	93.0	80.5	98.5	98.3	98.0	PI3K,HDAC
SkEpi:Alisertib (MLN8237)	40.8	29.5	39.0	37.8	31.2	28.3	Aurora Kinase
SkEpi:Entinostat (MS-275)	0.0	11.5	10.9	28.2	32.7	44.4	HDAC
SkEpi:AZ 960	5.5	19.7	21.6	71.5	71.4	64.7	JAK
SkEpi:Belinostat (PXD101)	22.6	0.0	12.8	55.9	41.6	39.6	HDAC
SkEpi:Mocetinostat (MGCD0103)	18.1	5.8	13.9	40.8	40.8	42.1	HDAC
SkEpi:Azacitidine	0.0	20.2	24.1	37.0	52.6	46.1	DNA Methyltransferase
SkEpi:Aurora A Inhibitor I	18.3	16.3	28.2	23.4	41.8	44.2	Aurora Kinase
SkEpi:Givinostat (ITF2357)	0.0	4.0	21.4	55.9	69.1	50.6	HDAC
SkEpi:Panobinostat (LBH589)	91.1	85.6	69.9	98.5	98.3	97.6	HDAC
SkEpi:TG101348 (SAR302503)	0.0	14.0	13.8	35.3	44.0	26.6	JAK
SkEpi:MK-5108 (VX-689)	0.0	4.6	14.7	54.6	40.9	48.8	Aurora Kinase
SkEpi:CUDC-101	26.0	12.7	30.9	54.7	34.3	65.1	EGFR,HER2,HDAC
SkEpi:PCI-24781 (Abexinostat)	8.4	3.9	23.3	54.5	63.5	53.3	HDAC
EzEpi:Trichostatin A	24.3	20.9	30.6	97.9	95.3	90.8	HDAC
EzEpi:Oxamflatin	26.4	0.7	21.8	55.9	29.9	43.2	HDAC

0.0	20.0	40.0	60.0	80.0	100.0

% of growth inhibition

Supplementary Table 3:
Inhibition scores of anti-cancer agents and epigenetic modulators against Calu-1 isogenic cells (p53$^{-/-}$, p53^{wt}, p53^{R158G}) at 0.1 and 1 µM of respective inhibitors.

Supplementary Table 4

Genes	fold change (log2)				ChIP-seq	
	$p53^{R158G}$ U / $p53^{wt}$ U	$p53^{R158G}$ T / $p53^{wt}$ T	$p53^{wt}$ T / $p53^{wt}$ U	$p53^{R158G}$ T / $p53^{R158G}$ U	$p53^{wt}$	$p53^{R158G}$
PCNA	-0.961	0.404	1.636	3.001		
POLA2	-0.949	0.192	1.614	2.756		
RFC4	-0.704	0.499	1.554	2.756		
RAD51	-0.87	-0.078	1.976	2.767		
TRAIP	-1.075	0.646	1	2.72		
USP1	-0.131	0.064	1.599	1.794		
POLE3	-0.168	0.08	1.504	1.752		
PSME3	0.06	-0.346	1.984	1.578		
CDK1	-0.302	0.423	0.938	1.662		
ANAPC7	-0.391	0.23	1.128	1.749		
PSMC4	-0.953	-0.351	1.355	1.957		
CDC7	-0.431	-0.854	2.537	2.114		
CYCS	-0.833	-1.114	2.425	2.144		
CHEK1	-1.379	-1.392	1.505	1.492		
TJP2	-0.919	0.638	0.526	2.082		
RBL1	-0.95	0.6	0.478	2.029		
MSH2	-1.033	0.734	-0.074	1.693		
POLE3	-0.08	1.253	0.183	1.516		
EXO1	-2.142	0.745	1.914	4.801		
ORC1	-1.964	0.318	1.411	3.693		
PMAIP1	-1.336	-2.121	2.44	1.655		
CDKN1A	-2.17	-2.701	2.131	1.601		
TNFRSF10B	2.006	-0.657	2.606	-0.057		
RPS27L	1.633	-1.123	1.878	-0.878		
MDM2	-0.258	-1.769	2.275	0.764		
GADD45A	0.352	-1.48	1.814	-0.018		
TDG	1.392	0.253	2.543	1.404		
ATF3	2.586	-1.578	4.887	0.723		

-3	-1	0	1	3	5

fold change (log2)

Supplementary Table 4:
Transcriptomic regulation (in fold changes) of wtp53 and p53R158G pre- and post- treatment (presented as the fold change of belinostat/cisplatin combination), as well as the respective p53 binding to TSS. Blue indicates ChIP binding of $p53^{wt}$; red indicates ChIP binding of $p53^{R148G}$.

Supplementary Table 5

Gene		Sequence 1	Sequence 2
BCL2	Primer	5'-AGCCAGGAGAAATCAAACAGAG-3'	5'-GATGACTGAGTACCTGAACCG-3'
	Probe	5'-/56-FAM/CAGGATAAC/ZEN/GGAGGCTGGGATGC/3IABkFQ/-3'	
BAX	Primer	5'-GCCACTCGGAAAAAGACCT-3'	5'-CGTCCACCAAGAAGCTGAG-3'
	Probe	5'-/56-FAM/ACATGGAGC/ZEN/TGCAGAGGATCATTGC/3IABkFQ/-3'	
BAK1	Primer	5'-CAGAAGAGCCACCACACG-3'	5'-CGACATCAACCGACGCTAT-3'
	Probe	5'-/56-FAM/TCAGAGTTC/ZEN/CAGACCATGTTGCAGC/3IABkFQ/-3'	
BAD	Primer	5'-CATCTGCGTTGCTGTGC-3'	5'-CCGGAGGATGAGTGACGA-3'
	Probe	5'-/56-FAM/TTTGTGGAC/ZEN/TCCTTTAAGAAGGGACTTCC/3IABkFQ/-3'	
CDKN1A	Primer	5'-GAGACTAAGGCAGAAGATGTAGAG-3'	5'-GCAGACCAGCATGACAGAT-3'
	Probe	5'-/56-FAM/TTCCTCTTG/ZEN/GAGAAGATCAGCCGG/3IABkFQ/-3'	
MDM2	Primer	5'-GTGCATTTCCAATAGTCAGCTAA-3'	5'-AGAAGGACAAGAACTCTCAGATG-3'
	Probe	5'-/56-FAM/TCACTCTCC/ZEN/CCTGCCTGATACACA/3IABkFQ/-3'	
PUMA	Primer	5'-CACCTAATTGGGCTCCATCT-3'	5'-ACGACCTCAACGCACAGTA-3'
	Probe	5'-/56-FAM/TGCTCCTCT/ZEN/TGTCTCCGCCG/3IABkFQ/-3'	
PMAIP1	Primer	5'-GAGCAGAAGAGTTTGGATATCAGA-3'	5'-GCAAGAACGCTCAACCGA-3'
	Probe	5'-/56-FAM/AGTTCAGTT/ZEN/TGTCTCCAAATCTCCTGAGTTG/3IABkFQ/-3'	
TRAIP	Primer	5'-TGCTGTCTCAAACCACTGAA-3'	5'-CACTATCTGCTCCGACTTCTTC-3'
	Probe	5'-/56-FAM/CGGCCACAC/ZEN/CTTCCACTTGC/3IABkFQ/-3'	
KAT6A	Primer	5'-CTCTGGCTTCTTTTCTCGGT-3'	5'-GTCTCTTTCCTGTTTACCTCCA-3'
	Probe	5'-/56-FAM/TTGCTGAAC/ZEN/CAATCCCCATCTGTAGTT/3IABkFQ/-3'	
KMT2D	Primer	5-'TGTGAAGGTGCTCTGATATGC-3'	5'-AGCTGCCACTCATGATCAAC-3'
	Probe	5'-/56-FAM/CTACAAACG/ZEN/GCCCCATACCCTGAA/3IABkFQ/-3'	
GADD45A	Primer	5'-GGAGATTAATCACTGGAACCCA-3'	5'-TGTACGAAGCGGCCAAG-3'
	Probe	5'-/56-FAM/ATCCATGTA/ZEN/GCGACTTTCCCGGC/3IABkFQ/-3'	
RAD51	Primer	5'-ACATTATCCAGGACATCACTGC-3'	5'-GCCATGTACATTGACACTGAG-3'
	Probe	5'-/56-FAM/ACCATACCT/ZEN/CTCAGCCACTGCC/3IABkFQ/-3'	
GAPDH	Primer	5'-GCGCCCAATACGACCAA-3'	5'-CTCTCTGCTCCTCCTGTTC-3'
	Probe	5'-/56-FAM/CCGTTGACT/ZEN/CCGACCTTCACCTT/3IABkFQ/-3'	
***TRAIP_2* (5' TSS)**	Primer	5'-TAGAATCGCCCGGAACTGAG-3'	5'-GCAGCCGCAAGGGAATAGAA-3'
***TRAIP_1* (3' TSS)**	Primer	5'-GCATGGTCCATCACTGTCCA-3'	5'-ATTGTGGTCCCATGCCAGAG-3'

Supplementary Table 5:
Sequences for primers and probes used for RT-qPCR and ChIP-qPCR.
