# Supplementary material for: Targeting codon 158 p53-mutant cancers via the induction of p53 acetylation
Source: Nat Commun. 2020 Apr 29;11:2086. doi: 10.1038/s41467-020-15608-y (PMC7190866; doi:10.1038/s41467-020-15608-y)
Supplement: Supplementary file 3 — Reporting summary [file 41467_2020_15608_MOESM3_ESM.pdf]

nature research

Corresponding author(s): Boon-Cher Goh & Li-Ren Kong

Last updated by author(s): Feb 28, 2020

Reporting Summary

Nature Research wishes to improve the reproducibility of the work that we publish. This form provides structure for consistency and transparency in reporting. For further information on Nature Research policies, see Authors & Referees and the Editorial Policy Checklist .

Statistics

For all statistical analyses, confirm that the following items are present in the figure legend, table legend, main text, or Methods section.

n/a	Confirmed	
☐	☒	The exact sample size (*n*) for each experimental group/condition, given as a discrete number and unit of measurement
☐	☒	A statement on whether measurements were taken from distinct samples or whether the same sample was measured repeatedly
☐	☒	The statistical test(s) used AND whether they are one- or two-sided<br>*Only common tests should be described solely by name; describe more complex techniques in the Methods section.*
☐	☒	A description of all covariates tested
☐	☒	A description of any assumptions or corrections, such as tests of normality and adjustment for multiple comparisons
☐	☒	A full description of the statistical parameters including central tendency (e.g. means) or other basic estimates (e.g. regression coefficient) AND variation (e.g. standard deviation) or associated estimates of uncertainty (e.g. confidence intervals)
☐	☒	For null hypothesis testing, the test statistic (e.g. *F*, *t*, *r*) with confidence intervals, effect sizes, degrees of freedom and *P* value noted<br>*Give P values as exact values whenever suitable.*
☒	☐	For Bayesian analysis, information on the choice of priors and Markov chain Monte Carlo settings
☒	☐	For hierarchical and complex designs, identification of the appropriate level for tests and full reporting of outcomes
☒	☐	Estimates of effect sizes (e.g. Cohen's *d*, Pearson's *r*), indicating how they were calculated

Our web collection on statistics for biologists contains articles on many of the points above.

Software and code

Policy information about availability of computer code

Data collection	NA
Data analysis	Molecular Dynamics analysis: pemed.CUDA module of the program Amber16 with the Amber 14SB force field (ff14SB), accelerated MD (aMD) implemented in AMBER16, simulation trajectories were visualized using VMD and figures were generated using Pymol.<br>Chiq-seq analyses: bowtie v2.1.053, MACS 2.0.936, MEME-ChIP54, HOMER (Hypergeometric Optimization of Motif EnRichment) 55 v4.5., ngs.plot, v2.61.<br>Gene expression (Ampliseq) analyses: Torrent Mapping Alignment Program (TMAP), R/Bioconductor package DESeq2.<br>Comet analyses: ImageJ software with OpenComet plugin (Gyori et al., 2014).<br>Image analyses (IF and IHC): ImageJ Software (1.51n) with Color Deconvolution plugin, and Particle Analyzer plugin.<br>FACS: FlowJo 7.5<br>Statistics: GraphPad 7

For manuscripts utilizing custom algorithms or software that are central to the research but not yet described in published literature, software must be made available to editors/reviewers. We strongly encourage code deposition in a community repository (e.g. GitHub). See the Nature Research guidelines for submitting code & software for further information.

Data

Policy information about availability of data

All manuscripts must include a data availability statement. This statement should provide the following information, where applicable:
- Accession codes, unique identifiers, or web links for publicly available datasets
- A list of figures that have associated raw data
- A description of any restrictions on data availability

The pan-cancer oncotated mutation annotation file were downloaded from GDAC (version 2016_01_28 (Cancer Genome Atlas Research, 2016)).

The dimeric structure of the p53 DNA Binding Domain (DBD) complexed to a small fragment of DNA (PDB ID 2AHI) was taken from the RCSB Protein Data Bank. The ChIP-seq and AmpliSeq datasets generated during the current study have been deposited in GEO with the Accession number "GSE129027" and could be accessed at this link: https://www.ncbi.nlm.nih.gov/geo/query/acc.cgi?acc=GSE129027. The unprocessed immunoblots are provided as a Source Data file. Additional data could be provided by the correspondence upon request.

Field-specific reporting

Please select the one below that is the best fit for your research. If you are not sure, read the appropriate sections before making your selection.

[x] Life sciences [ ] Behavioural & social sciences [ ] Ecological, evolutionary & environmental sciences

For a reference copy of the document with all sections, see nature.com/documents/nr-reporting-summary-flat.pdf

Life sciences study design

All studies must disclose on these points even when the disclosure is negative.

Sample size	No sample size calculation was applied in this study. Experiments were performed independently in an unbiased manner. Experiments were repeated 2-3 times (as indicated in respective figure legends). Findings were considered as significant and relevant when consistent observations were made.
Data exclusions	Western blot data for overexpression study was omitted when plasmid transfection failed as protein overexpressions were not comparable across isogenic clones.
Replication	All in vitro studies were performed independently by at least two researchers to ensure reproducibility. All In vivo investigation was conducted by an independent research team, with the tumour growth propagated at least twice before experimentations. All data collected were included in the analyses.
Randomization	Animals used in this study were randomised into various treatment groups based on their body weight. Each group contained equal distribution of mice with comparable weight. All in vitro studies are randomized.
Blinding	Investigators were blinded during data acquisition. All images were anonymised prior to analyses. Researchers handling animal work only shared their findings upon completion of data collection.

Reporting for specific materials, systems and methods

We require information from authors about some types of materials, experimental systems and methods used in many studies. Here, indicate whether each material, system or method listed is relevant to your study. If you are not sure if a list item applies to your research, read the appropriate section before selecting a response.

Materials & experimental systems

n/a	Involved in the study	
[ ]	[x]	Antibodies
[ ]	[x]	Eukaryotic cell lines
[x]	[ ]	Palaeontology
[ ]	[x]	Animals and other organisms
[x]	[ ]	Human research participants
[x]	[ ]	Clinical data

Methods

n/a	Involved in the study	
[ ]	[x]	ChIP-seq
[ ]	[x]	Flow cytometry
[x]	[ ]	MRI-based neuroimaging

Antibodies

Antibodies used

Antibodies used for immunoblotting include: PARP (#9542), cleaved PARP (#5625), caspase 3 (#9662), caspase 7 (#9492), caspase 9 (#9502), acetyl-H3K23 (#9674), acetyl-H3K9K14 (#9677), total H3 (#9715), phospho-p53 Ser15 (#9286), acetyl-p53 Lys 382 (#2525), acetyl-p53 Lys379 (#2570), total p53 (#9282), p21 (#2947), γH2AX (#9718), phopho-IκB Ser32 (#2859), total IκB (#9247), total p65 (#8242), phoshph-TRAF2 Ser11 (#13908), total TRAF2 (#4724), c-IAP1 (#7065), c-IAP2 (#3130), α-tubulin (#2125), and horseradish peroxidase (HRP)-conjugated β-actin (#5125) antibodies as well as secondary anti-mouse (#7076) and –rabbit (#7074) HRP-conjugated secondary antibodies were obtained from Cell Signaling Technologies; acetyl-H4 (#06-866) antibody was from Merck Millipore; TRAIP (#ab80170), TATA-box binding protein (#ab818) were from Abcam; DDK (#TA180144) antibody was from Origene. All antibodies were used at 1:2000 dilution.

Immunofluorescence were done with acetyl-p53 (1:500, Cell Signaling, #2570), p-p53 (1:500, Cell Signaling, #9286), or NF-κB p65 (1:1000, Cell Signaling, #8242) for 4 hours at 4oC, followed by incubation with Alexa Fluor-488 or -594 secondary antibodies (1:1000, Molecular Probes, Life Technologies, A11034 and R37121).

For IHC, slides were incubated with primary antibodies [NF-κB p65 (1:1000, Cell Signaling, #8242), cleaved caspase 3 (1:1000, Cell Signaling, #9661), Ki67 (1:1000, Abcam, #ab15580).

Validation	All used antibodies were validated by the manufacturer for the indicated purposes (WB, IF, IHC, ChIP), and reviewed positively by peers. Antibodies selected are selective to human, and have to be validated in house with representative imaged provided in the datasheet provided

Eukaryotic cell lines

Policy information about cell lines

Cell line source(s)	Cancer cell lines (Calu-1, ChaGo-k-1, HCC70, H441, H520, H596, H661, H747, H1417, H2170, MDA-MB-468, SK-BR-3, SK-MES-1) and lung fibroblast cells (MRC-5) were obtained directly from the American Type Culture Collection (ATCC), while others (A549, BT-549, H1975, MIA-Paca-2, PANC-1) were provided by Ashok Venkitaraman (MRC Cancer Unit, Cambridge, UK). Cultures were maintained in a humidified 37°C incubator, in culture medium (according to ATCC's recommendation) supplemented with 10% fetal bovine serum, 2mM L-glutamine, 100 µg mL-1 streptomycin and 100 U mL-1 penicillin. Paired HCT116 p53-/- and p53+/+ cell lines were obtained from Bert Vogelstein (The John Hopkins Medical School, Baltimore, MD) as a gift. Isogenic Calu-1 cells stably expressing pcDNA3.1 vector, pcDNA3.1-p53wt, pcDNA3.1-p53R158G, pcDNA3.1-p53R158G(DBD, K-A), pcDNA3.1-p53R158G(CT, K-A), pcDNA3.1-p53R158G(K20A) plasmids respectively, were generated by transfection and single-cell selection.
Authentication	All cell lines were authenticated using Promega GenePrint STR method
Mycoplasma contamination	Mycoplasma contamination test is performed regularly (6-9 months) in our lab. Newly acquired cell lines were tested before experimentation. Only cell lines free of mycoplasma contamination were used in this study.
Commonly misidentified lines (See ICLAC register)	No commonly misidentified cell line was used in this study.

Animals and other organisms

Policy information about studies involving animals; ARRIVE guidelines recommended for reporting animal research

Laboratory animals	All in vivo xenograft studies were established and maintained in 8- to 10-week-old female SCID mice.
Wild animals	No wild animals were used in this study.
Field-collected samples	No field-collected samples were used in this study.
Ethics oversight	We adhered to the Institutional Animal Care and Use Committee (IACUC) guidelines on animal use and handling

Note that full information on the approval of the study protocol must also be provided in the manuscript.

ChIP-seq

Data deposition

- [x] Confirm that both raw and final processed data have been deposited in a public database such as GEO.
- [x] Confirm that you have deposited or provided access to graph files (e.g. BED files) for the called peaks.

Data access links *May remain private before publication.*	the raw files have been uploaded and deposited to GEO. GEO superSeries: GSE129027.
Files in database submission	bigwig files, raw fastq files
Genome browser session (e.g. UCSC)	No longer applicable.

Methodology

Replicates	pair-end reads were performed on each samples (n=1). Identified peaks were validated with ChIP-qPCR using the same DNA samples for ChIP-Seq as well as independent ChIP samples.
Sequencing depth	At least 16 million 51-bp long reads were mapped to hg19 using bowtie v2.1.053 with parameters -N 1 –sensitive -p 2 –no-unal.
Antibodies	p53 and rabbit IgG antibodies (Cell Signaling, #9282 and #2729)
Peak calling parameters	Peaks were identified by MACS 2.0.936 using a maximum of 2 reads per unique position and otherwise default parameters.
Data quality	Only highly enriched (enrichment over background ≥5-fold, pileup ≥25) and highly significant (q-value<0.01) peaks were shortlisted from the analysis.

Software	bowtie v2.1.053, MACS 2.0.936, MEME-ChIP54, HOMER (Hypergeometric Optimization of Motif EnRichment) 55 v4.5., ngs.plot, v2.61.

Flow Cytometry

Plots

Confirm that:

- [x] The axis labels state the marker and fluorochrome used (e.g. CD4-FITC).
- [x] The axis scales are clearly visible. Include numbers along axes only for bottom left plot of group (a 'group' is an analysis of identical markers).
- [x] All plots are contour plots with outliers or pseudocolor plots.
- [x] A numerical value for number of cells or percentage (with statistics) is provided.

Methodology

Sample preparation	Cell lines were treated with the indicated treatment. Live cells were stained with Annexin V-APC, and fixed cells were stained with propidium iodide.
Instrument	DB LSRII
Software	BD FACSDiva for acquisition and FlowJo for analyses
Cell population abundance	at least 10,000 cells
Gating strategy	Gating was peformed with the control cells (in our case DMSO treated cells). Gating strategies were either demonstrated in the figure, or in the Supplementary Information files.

- [x] Tick this box to confirm that a figure exemplifying the gating strategy is provided in the Supplementary Information.
